# Efficacy of Acupuncture for Parkinson's Disease Anxiety: Two-Stage Protocol for a Randomized Controlled Clinical Trial

**DOI:** 10.1155/2022/5180193

**Published:** 2022-02-09

**Authors:** Jing-Qi Fan, Zi-Qiao Xu, Yuan-Yuan Chen, Wei-Jing Lu, Xiao-Yan Xie, Yu-Ting Wang, Li-Xing Zhuang

**Affiliations:** ^1^Guangzhou University of Chinese Medicine, Guangzhou 510000, Guangdong, China; ^2^The First Affiliated Hospital of Guangzhou University of Chinese Medicine, Guangzhou 510000, Guangdong, China

## Abstract

Parkinson's disease anxiety (PDA) is a nonmotor symptom of Parkinson's disease (PD) that is often neglected. PDA poses a far-reaching challenge to the treatment of PD. Acupuncture could be successful in the treatment of PDA. However, the evidence for this is still limited. We propose a two-stage clinical trial. In stage 1, a total of 70 volunteers with PDA will be randomly assigned to either acupuncture (manual acupuncture) or control group (sham acupuncture) in a 1 : 1 ratio. Treatments will be performed for four weeks. The change in the Hamilton Rating Scale for Anxiety (HAMA) score from baseline to week 4 and week 12 will be the primary outcome. The levels of adrenocorticotropic hormone (ACTH), cortisol (CORT), serotonin (5-HT), and corticotropin-releasing factor (CRH) in the patients' serum and the scores on the Hoehn–Yahr Rating Scale and the Unified Parkinson's Disease Rating Scale (UPDRS) will all be considered among the secondary outcomes. Participants will be followed up until week 12. In stage 2, a total of 82 volunteers with PDA will be randomly assigned to either an acupuncture (manual acupuncture) or a control group (anti-Parkinson drugs only) in a 1 : 1 ratio. HAMA score will be the primary outcome. Universality, feasibility and cost effectiveness, Hoehn–Yahr Rating Scale, UPDRS, and serological indicators will be secondary outcomes. Participants will be followed up until week 4. The statistical analysis will include all the allocated individuals. The First Affiliated Hospital of Guangzhou University of Traditional Chinese Medicine's Research Ethical Committee authorized this procedure, and the trial is registered with ChiCTR2100047253.

## 1. Introduction

Although Parkinson's disease (PD) is typically characterized by the presence of rest tremors, bradykinesia, and rigidity, it is also a complex disease with many nonmotor manifestations [1]. Parkinson's disease anxiety (PDA) is a nonmotor manifestation of Parkinson's disease (PD) that is characterized by a persistent sense of worry, muscle tension, inability to concentrate, headache, and insomnia [2]. PDA is most likely an unintended consequence of PD treatment, etiology, or symptom, which is frequently overlooked and untreated due to its classification as a nonmotor symptom. However, approximately 31% of patients with PD are diagnosed with PDA [3], which aggravates the progression of the disease [4, 5] and results in symptom fluctuations [6]. PDA poses a significant challenge to the treatment of PD, endangers patients' lives, and aggravates the motor disorder, imposing a significant financial burden on families and society.

Pharmacology and psychotherapy are the most commonly used treatments for PDA [5, 7]. However, no clinical studies have been conducted to confirm the efficacy of these methods [8]. Unwanted side effects, decreased cognitive ability, balance complications, and sedation have all been linked to pharmacological treatments, putting patients at risk of falling [9]. Some anti-Parkinson and antianxiety medications cannot be used simultaneously because they may aggravate Parkinson's disease [10]. Psychotherapy has few side effects but takes a long time and is costly for patients [11]. It is critical to avoid increasing the financial burden on patients while ensuring the effectiveness and feasibility of the treatment. There is a need for more appropriate complementary therapies for the treatment of PDA. Acupuncture is one of the most popular complementary and alternative medicine treatment methods due to its short duration, minimal side effects, low cost, high acceptance, and significant curative effect [9].

Furthermore, some studies have shown that acupuncture treatment has integrative effects in the treatment of PD and anxiety [12–16]. However, to date, there have been no randomized controlled trials on the efficacy of acupuncture in the treatment of PDA. In the first stage of this trial, we hope to gather evidence on the efficacy and mechanism of acupuncture in the treatment of PDA. It cannot be verified that acupuncture is suitable for popular application for PDA treatment in the clinic solely on the basis of confirming the curative effect, so the clinical application value of acupuncture must be further proven. This is the second stage trial that will assess the clinical efficacy, compliance, universality, and expense of acupuncture for PDA. We hypothesize that acupuncture can significantly reduce anxiety symptoms in PD patients while also ensuring cost effectiveness and compliance. We intend to demonstrate that acupuncture can be used as a complementary therapy with high therapeutic potential for PDA.

## 2. Materials and Methods

### 2.1. Trial Design

This study will be a two-stage clinical trial for Chinese patients with PDA. The first stage will be a randomized single-blinded controlled clinical trial with a sham acupuncture control, which aims to evaluate the efficacy of acupuncture. The second stage will be a randomized controlled clinical trial with blank control, which aims to analyze the efficacy, cost effectiveness, and compliance feasibility of acupuncture for PDA. The trial will be conducted at Guangzhou University of Chinese Medicine's First Affiliated Hospital in China.

Ethics committees at the First Affiliated Hospital of Guangzhou University of Chinese Medicine have accepted the study protocol, which will follow the Declaration of Helsinki. It has been registered with Current Controlled Trials (ChiCTR2100047253).

**Figure 1:**
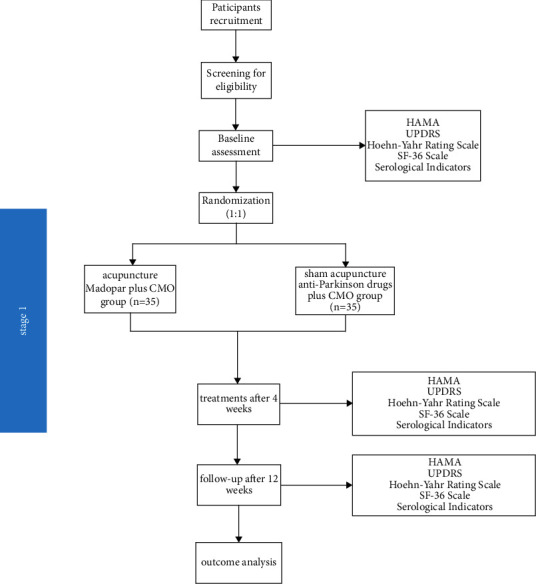
Flowchart of stage 1 of the trial.

Stage 1 will be a randomized clinical trial using a sham acupuncture control.  Figure 1 depicts the flowchart for the first step.

### 2.2. Diagnostic Criteria

The clinical diagnostic criteria for PD, as revised by the International Association of Dyskinesia in 2015, will be used as the diagnostic criteria [17]. At present, there is no internationally recognized diagnostic standard for PDA. In previous studies, the diagnostic criteria of PD and anxiety disorder were often combined. Patients with idiopathic PD will be included irrespective of their disease stage or current anti-Parkinson medication if they show the presence of clinically relevant anxiety symptoms, as determined by the Hamilton Rating Scale for Anxiety (HAMA) (score range: 14 to 29) [18].

### 2.3. Inclusion Criteria for Participants

The inclusion criteria are as follows:

1. Idiopathic PD diagnosis
2. Hoehn and Yahr staging scale stages 1 to 4
3. HAMA scale score range of 14 to 29
4. Ability to provide written informed consent
5. Men or women aged 35 to 80 years

### 2.4. Exclusion Criteria for Participants

The exclusion criteria are as follows:

1. Significant neurologic, renal, cardiovascular, or hepatic impairment
2. Any disease that can result in Parkinson's syndrome or other conditions that are suspected to be related to the patient's symptoms
3. Significant cognitive impairment as defined by the score of the Montreal Cognitive Assessment (MOCA) of <23
4. No significant response to high-dose levodopa therapy
5. Medical or psychological condition that makes participation in the study challenging
6. Use of or dependence on drugs or alcohol that could affect participation in the study
7. Within 30 days of treatment initiation, exposure to the study drug or acupuncture
8. Allergy or intolerance to acupuncture
9. Inability to adhere to the study protocol as determined by the investigator

### 2.5. Sample Size

The sample size was calculated using the primary outcome, which is a shift in the HAMA score from baseline. According to preliminary results, the mean HAMA score in patients who received acupuncture combined with anti-Parkinson drugs was 15.3, while it was 13.2 in patients who received the sham acupuncture with anti-Parkinson drugs alone. Using SPSS Statistics 26.0 (IBM SPSS Statistics Inc., Chicago, USA), we determined that a sample comprising 62 patients (31 in each group) would have a power of 80% or more to identify a two-sided significance level of 5%. The overall sample size needed for the study is 70 (35 in each group), anticipating a 10% dropout rate. If the patient cannot adhere to a course of acupuncture, patient's condition suddenly worsens during the treatment process, or the treatment plan of anti-Parkinson drugs needs to be changed, the acupuncture regimen will be stopped.

### 2.6. Randomization

Participants will be allocated to two intervention groups at random in a ratio of 1 : 1 to receive acupuncture plus anti-Parkinson drugs or anti-Parkinson drugs with sham acupuncture. A randomization sequence will be generated by a statistician not participating in this trial using SPSS Statistics 26.0 (IBM SPSS Statistics Inc., Chicago, USA). The assignment sequences will be concealed in sequentially numbered, sealed, and opaque envelopes to ensure sequence confidentiality.

### 2.7. Blinding

In stage 1, we will use the single-blinded method. We will treat patients in the acupuncture and the control group with real acupuncture and sham acupuncture, respectively. The acupuncture time and acupoints of the control group will be the same as those in the acupuncture group. Both real and sham acupuncture will use the same base and sleeve, which is consistent in appearance, and the patients will both have a sense of penetration. Real acupuncture and sham acupuncture will be numbered according to a random figure which will be blinded to patients. Patients will not be able to distinguish the treatment they receive; however, the operating doctor will know the kind of needle they use, thereby achieving a single-blind outcome.

**Figure 2:**
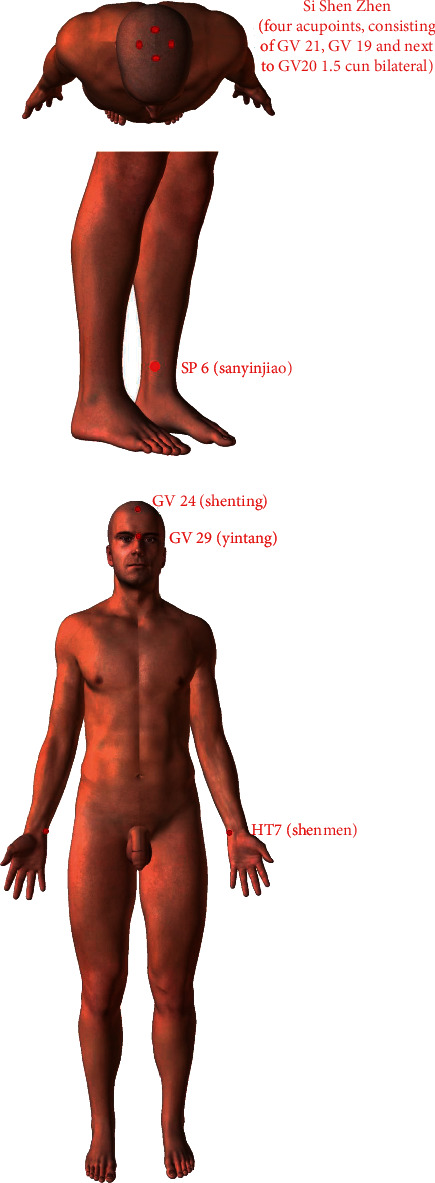
Selection and location of the acupoints for acupuncture.

**Figure 3:**
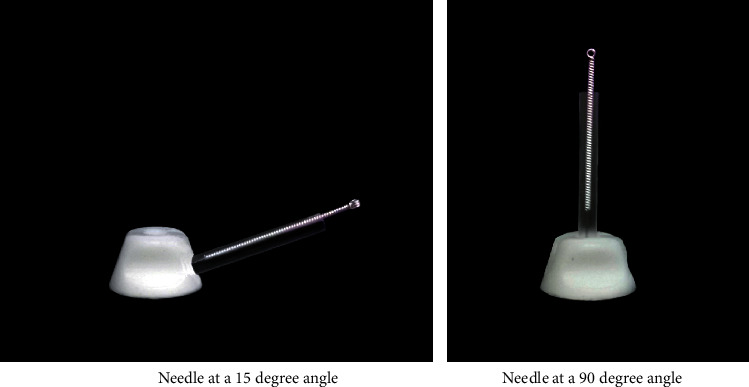
Main structure of the sham acupuncture needle.

**Figure 4:**
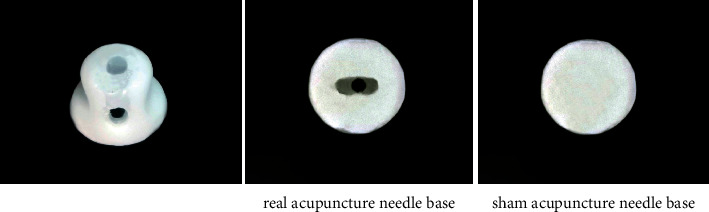
Acupuncture needle base.

**Figure 5:**
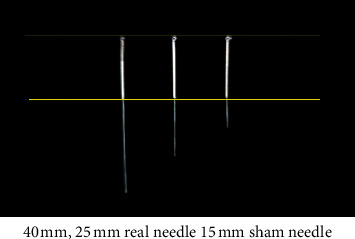
Real needle and sham needle.

The selection and location of the acupoints are shown in Figure 2. In this study, the *Si Shen Zhen* and *shen ting* acupoints will be penetrated at an angle of 15-degree angle. At present, other types of sham needles cannot achieve this purpose. Therefore, our team has independently developed a sham needle, which penetrates in all directions on the premise of achieving fixation. At present, this technology has obtained a patent under the authorization of the State Intellectual Property Office of China, with the authorization number 202121352221.7. Figures 3–5 show the structure of the sham acupuncture needle.

## 3. Interventions

### 3.1. Acupuncture Group

Participants assigned to the acupuncture group will receive twelve 30 min sessions of acupuncture (once every other day, four times a week for four weeks) with clinical monitoring only (CMO) and maintain their original dosage of anti-Parkinson drugs. If the dosages need to be increased or decreased during the trial to relieve symptoms, the change will be recorded on the case report form. Patients in the intervention group will receive needling at traditional acupuncture points, including GV 24 (*shen ting*), GV 29 (*yin tang*), bilateral HT7 (*shen men*), bilateral SP 6 (*san yin jiao*), and *Si Shen Zhen* (four acupoints, consisting of GV 21, GV 19, and 1.5 cun next to GV 20 bilaterally, with the width of the patient's thumb joint being taken as one cun). The acupoints' positions are described by the National Standard of the People's Republic of China's (GB/T 12346–2006) standard for the name and location of the acupoints, which was established in 2006. Acupuncture will be performed with disposable, sanitized stainless steel needles (Tianxie, Suzhou Medical Appliance Factory, Suzhou, China; 0.25 × 25 mm, 0.25 × 40 mm). GV 24 (*shen ting*), GV 29 (*yin tang*), and *Si Shen Zhen* adopt 0.25 × 25 mm acupuncture needles with a 45-degree angle. Bilateral HT7 (*shen men*) and bilateral SP 6 (*san yin jiao*) will use 0.25 × 40 mm acupuncture needles with a 90-degree angle. The selection and location of the acupoints are shown in Figure 2. After routine disinfection, the acupuncturist inserts the needles into the corresponding acupoints and stimulates all the needles by lifting, twirling, and thrusting to obtain the *de qi* sensation (a combination of feelings, such as numbness, soreness, and heaviness).

CMO will involve providing patients with general educational leaflets on dealing with anxiety that were retrieved from web portals of the French and Netherlands psychiatric associations.

### 3.2. Control Group

Participants assigned to the control group will receive twelve 30 min sessions of acupuncture with sham acupuncture needles (once every other day, four times a week for four weeks) with CMO and maintain the original dosage of their anti-Parkinson drugs. If dosages need to be increased or decreased during the trial to relieve symptoms, the change will be recorded on the case report form. For GV 24 (*shen ting*), GV 29 (*yin tang*), and *Si Shen Zhen*, 0.25 × 25 mm acupuncture needles with a 45-degree angle will be used. For Bilateral HT7 (*shen men*) and bilateral SP 6 (*san yin jiao*), 0.25 × 40 mm acupuncture needles with a 90-degree angle will be used. In this study, some acupoints need to be penetrated at an angle of 15°. This acupuncture tool consists of a base sticking to the skin and a sleeve. In particular, there are two types of bases in this acupuncture tool, one is hollow while the other is sealed by the adhesive layer. Specifically, we will paste this special acupuncture tool on the skin first. In the acupuncture group, we will put acupuncture needles through the cannula and pierce them into the skin through the perforated base to achieve the acupuncture effect. However, when used in the control group, a special placebo needle will be used to pass through the cannula and pressed against the adhesive layer and the skin, and a slight pressure will be applied to ensure a placebo effect similar to acupuncture. In this way, we hope to achieve a single-blind effect on the patients to verify the placebo effect in acupuncture. Figures 3–5 show the structure of the sham acupuncture needle.

### 3.3. Outcome Measurements

The primary outcome measure will be the HAMA score. The secondary outcome will be the Hoehn–Yahr Rating Scale; Unified Parkinson's Disease Rating Scale (UPDRS); and blood serum levels of adrenocorticotropic hormone (ACTH), corticotropin-releasing factor (CRF), cortisol (CORT), and serotonin (5-HT). The serological indexes will be measured by enzyme-linked immunosorbent assay (ELISA). The timeline of the outcome measurements is shown in Table 1.

### 3.4. Primary Outcome

The change in the HAMA score from baseline will be the primary outcome, measured at baseline and week 4. We will also use this scale during the follow-up period at week 12 to assess the anxiety status of the patients after they leave the group. HAMA is one of the first rating scales used to measure the severity of perceived anxiety symptoms and is often used in clinical trials. It consists of 14 symptomatic definition elements that reflect psychological and physical symptoms. Each item is rated on a scale of 0 to 4, for a total possible score of 56. Mild anxiety is indicated by a score of 17–24, whereas severe anxiety is indicated by a score of 25–30.

### 3.5. Secondary Outcome Measurement

#### 3.5.1. Blood Serum Index

Deficiency of dopamine in PD suppresses the synthesis and release of 5-HT by neurons and thereby aggravates the anxiety levels in patients with PD [19]. Therefore, 5-HT serum levels may reflect the anxiety level of patients with PDA. When patients are anxious, the hypothalamus discharges CRF to induce the secretion of ACTH in the anterior pituitary gland. ACTH activates the adrenal cortex to discharge CORT simultaneously. In addition, CORT has a negative feedback effect on CRF and ACTH secretion. 5-HT plays a crucial role in controlling the hypothalamic-pituitary-adrenal axis (HPA). Therefore, the levels of CORT and ACTH can reflect the functional status of the HPA, and their measurement can thereby serve to assess anxiety in patients. We will measure the serological indicators CRF, ACTH, CORT, and 5-HT at baseline and at week 4 and week 12 of the trial.

#### 3.5.2. UPDRS and the Hoehn–Yahr Rating Scale

In addition to the assessment of patient anxiety, PD-associated nonmotor and motor symptoms are also a focus of our study. Hoehn–Yahr Rating Scale and UPDRS will therefore be employed in this study. The clinical assessment of PD mainly relies on the UPDRS. This scale, which provides cardinal evidence for determining the clinical classification, has been applied for 20 years as a comprehensive assessment tool to monitor disease progression and evaluate the therapeutic efficacy of PD treatments. The instrument assesses four aspects: (1) mood, mental status, and behavior; (2) daily life activities; (3) motor evaluation; and (4) problems of therapy. Based on the total score, PD can be divided into five grades: normal, mild, moderate, severe, and extremely severe. The Hoehn–Yahr Rating Scale, another scale applied in this trial, has been used for the staging of functional disability linked with PD. It describes the progression of PD-related symptoms through various stages, thus allowing us to assess the intensity of the disease. The above two scales will be completed under the guidance of professional personnel at baseline and weeks 4 and 12.

## 4. Quality of Life Assessment

### 4.1. SF-36 Scale

The SF-36 scale mainly measures eight items involving both physical and mental aspects and will be used to reflect the patient's quality of life over the previous 4 weeks. Every item is scored on a scale of 1 to 100; a greater score represents a better quality of life. The SF-36 scale is useful in predicting disease progression. The scale will be completed under the guidance of professional personnel at baseline and weeks 4 and 12.

### 4.2. Safety Evaluation

During the acupuncture of the participants, acupuncture operators will truthfully record adverse reactions, such as dizziness, broken needles, hematoma, and infection. At the same time, the operators will record and evaluate the treatment for adverse reactions. If patients drop out of the trial owing to adverse reactions, the reason for dropout and the last treatment time will be recorded, and the assessment items that can be completed before the end of the trial will be completed. After the end of the trial, the patient data will be collected and appropriately stored. Dropouts owing to adverse reactions will be included in a statistical analysis of adverse reactions.

## 5. Statistical Analysis

To ensure the integrity and accuracy of data, two statisticians will independently perform statistical analyses using Statistics 26.0 (IBM SPSS Statistics Inc., Chicago, USA), establish the database, and provide proofreading for logic. Enumeration data will be expressed as percentages, whereas measurement data will be expressed as mean ± standard deviation. A normality test will be performed for the measurement data. For variables that are determined to be regularly distributed, a *t*-test will be employed, and for those that are not, a rank-sum test will be utilized. The rank-sum test and nonparametric test will be employed for graded data, and the chi-square test will be applied to counted data. Repeated-measures analysis of covariance (ANCOVA) will be used for the UPDRS, Hoehn–Yahr, HAMA, and SF-36 scales. The rank-sum test will be applied to compare efficacy. Serological index values will be analyzed using covariance. Two-sided tests will be used for all hypothesis testing, with *P* < 0.05 as the standard of statistical significance.

**Figure 6:**
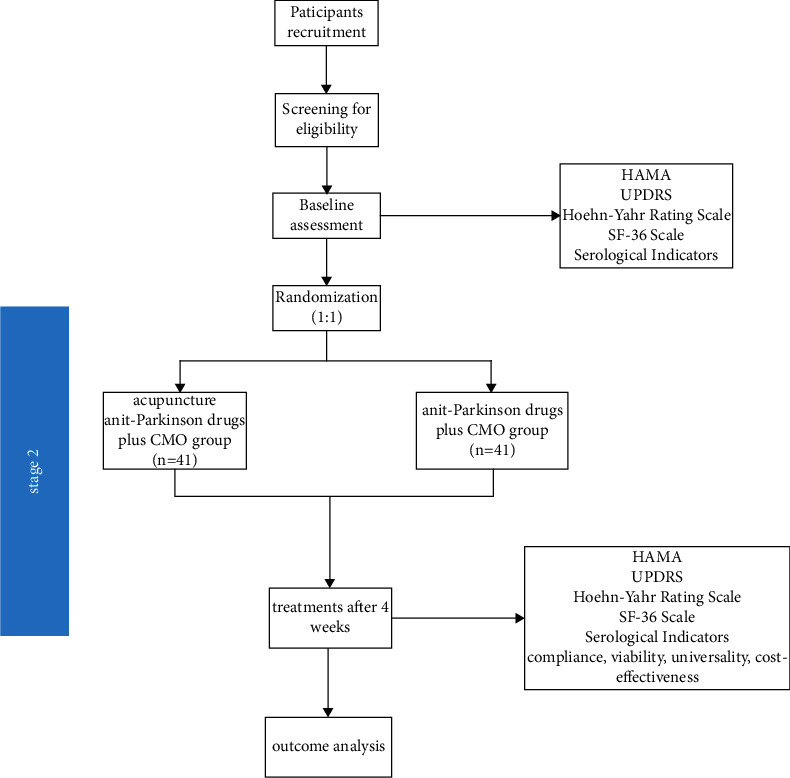
Flowchart of stage 2 of the trial.

Stage Two: if the efficacy of acupuncture with anti-Parkinson drugs for PDA is found to be better than that of the sham acupuncture, excluding the possible placebo effect of acupuncture, stage 2 will be conducted to assess the compliance, viability, universality, and cost effectiveness of acupuncture for PDA.  Figure 6 depicts a flowchart of the second step.

Diagnostic criteria:
    The diagnostic criteria for stage 2 are the same as those for stage 1
Participant inclusion criteria:
    The inclusion criteria for stage 2 are the same as those for stage 1
Participant exclusion criteria:
    The exclusion criteria for stage 2 are the same as those for stage 1

### 5.1. Sample Size

The sample size was calculated using the primary outcome, which is the shift in HAMA score from baseline. According to preliminary results, the mean HAMA score in patients who received acupuncture combined with anti-Parkinson drugs was 15.8, while it was 13.9 in patients who received anti-Parkinson drugs alone. According to PASS 15.0, a sample size comprising 74 patients (37 in each group) would have a power of 80% or more to identify a two-sided significance level of 5%. The overall sample size needed for the study is 82 (41 in each group), anticipating a 10% dropout rate. If a patient cannot adhere to a course of acupuncture, patient's condition suddenly worsens during the treatment process, and the treatment plan of anti-Parkinson drugs needs to be changed, the treatment will be stopped.

#### 5.1.1. Randomization

Patients will be randomly allocated in a 1 : 1 ratio to two intervention groups to receive acupuncture in combination with anti-Parkinson drugs or anti-Parkinson drugs alone. A randomization sequence will be generated by a statistician not participating in this trial, using SPSS Statistics 26.0 (IBM SPSS Statistics Inc., Chicago, USA). The assignment sequences will be concealed in sequentially numbered, sealed, opaque envelopes to ensure sequence confidentiality.

#### 5.1.2. Blinding

A single-blind method will be applied during stage 1 experiment to determine whether acupuncture has a placebo effect. In stage 2, considering the particulars of the acupuncture operation and the difficulty of implementing a blind method, both the participant and the physician will unavoidably know the treatment items delivered during the study. However, the outcome evaluators and statisticians will be blinded and not participate in the participant treatment process. Thus, bias in the research results would be reduced considerably.

#### 5.1.3. Setting

In stage 2, participants will be recruited from patients admitted to the First Affiliated Hospital of Guangzhou University of Traditional Chinese Medicine.

### 5.2. Interventions

#### 5.2.1. Acupuncture Group

Interventions for the acupuncture group in stage 2 will be the same as those in stage 1.

#### 5.2.2. Control Group

Participants in the control group will be required to obtain CMO and maintain the original dosage of their anti-Parkinson drugs. If the dosages need to be increased or decreased during the trial to relieve symptoms, the change will be recorded in the case report form.

#### 5.2.3. Outcome Measurements

HAMA score will be the primary outcome. The primary outcomes will be measured at baseline and week 4. Moreover, universality, feasibility, cost effectiveness, Hoehn–Yahr Rating Scale score, UPDRS score, and serological indicators will be considered the secondary outcomes. Hoehn–Yahr Rating Scale score, UPDRS score, and serological indicators will be measured at baseline and week 4. Universality, feasibility, and cost effectiveness will be measured at week 4. In addition, the universality and feasibility study will employ questionnaires administered by the physicians, and cost effectiveness will be calculated as the ratio of HAMA score improvement to treatment cost. Furthermore, side effects will be assessed during the trial. The timeline of the outcome measurements is shown in Table 2.

## 6. Parkinson's Disease Rating Scale

### 6.1. Statistical Analysis

All data will be analyzed using intention-to-treat (ITT) analysis and the per-protocol set (PPS) for coherence. As in stage one, normality tests will be performed on the measurement data. A *t*-test will be performed for variables found to be normally distributed, and the rank-sum test will be used for those not normally distributed. The rank-sum test and nonparametric test will be used for the graded data, and the chi-square test will be applied to counted data. Repeated-measurements ANOVA will be used for the UPDRS, Hoehn–Yahr, HAMA, and SF-36 scales. The rank-sum test will be used to compare efficacy.

### 6.2. Ethical Issues in Stages 1 and 2

The trial will be conducted according to the guidelines of the Declaration of Helsinki.

The study was approved by the Ethics Committee of the First Affiliated Hospital of Guangzhou University of Traditional Chinese Medicine (No. K [2021] 021). All the participants will be asked to provide informed consent before participating in the trial.

### 6.3. Quality Control in Stages 1 and 2

A research workbook will be established. All the investigators will be trained and tested for conformance with preset standards before the beginning of each stage. Each participant will have a fixed physician and time points for treatment to ensure consistency in the intervention effect. Since the use of anti-Parkinson drugs will affect the physician's evaluation of patient symptoms, the participants will be evaluated for PD symptoms 4 hours after taking their medication.

All acupuncturists participating in this project have received formal education at Traditional Chinese Medicine universities, obtained physician qualification certificates, and possess more than 3 years of clinical experience in acupuncture.

## 7. Discussion

Anxiety is more common in PD patients than in the general population [20]. PDA may be related to L-DOPA treatment, dopamine deficiency etiology, and complex PD symptoms.

**Figure 7:**
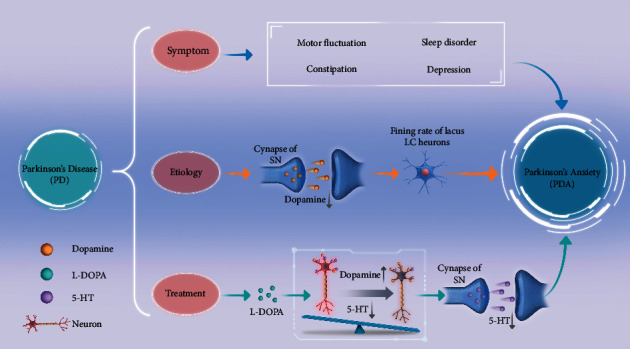
The relationship between PD and PDA.

The symptoms of PD are complex. In addition to motor symptoms, there are a series of nonmotor symptoms, such as sleep disorder, constipation, and depression. These complex symptoms seriously affect the quality of life of patients, resulting in emotional burden and provoking the development of disorders such as anxiety. Deficiency of dopamine in PD results in increased firing rate of the locus coeruleus (LC), which suppresses the synthesis and release of the 5-HT neurons and thereby aggravates the anxiety levels in patients with PD. However, excessive dopamine intake can easily affect the balance between dopamine and 5-HT, resulting in PDA. The decrease of endogenous dopamine in the striatum of PD patients and the excessive intake of dopamine during long-term drug use easily cause the occurrence of PDA. PDA may be an adverse outcome of disease progression in PD.  Figure 7 shows the possible pathogenesis of PDA and the relationship between PD and PDA [21, 22]. Relevant studies proved that acupuncture can supplement dopamine by maintaining the stability of dopamine in the substantia nigra striatum so as to avoid hyperactivity or lack of dopamine [23].

At present, anxiety is often neglected as one of the nonmotor symptoms of PD, and there is no drug for the treatment of PDA; hence, antianxiety drugs and antidepressant drugs are mainly used. However, antidepressant drugs, such as serotonin reuptake inhibitors (SSRI), combined with anti-Parkinson drugs, such as monoamine oxidase B inhibitors (MAO-BI), can easily aggravate tremor symptoms [24]. There is a lack of clinical evidence regarding the role of antianxiety drugs in PDA; only anecdotal evidence exists. Patients with anxiety benefit from more effective and safer forms of complementary and alternative interventions, such as acupuncture [25]. Several related studies have confirmed that acupuncture can improve the nonmotor symptoms of PD [9, 16]. Acupuncture for patients with PDA has not been intensively investigated. We will also investigate the mechanism of action of acupuncture on PDA in terms of the HPA, which is the center of the stress response, and 5-HT in stage 1. CRH, ACTH, and CORT are interrelated and interact within the HPA. When the human body is stimulated by anxiety, CRH can enter the anterior pituitary in the blood of the portal system and stimulate it to release ACTH, resulting in an increase in CORT secretion [26]. Le et al. [27] demonstrated that electric acupuncture was associated with a reduction in depressive symptoms via adjustment of HPA function [27]. Previous animal experiments have confirmed that acupuncture is effective in regulating the levels of HPA-related hormones [28, 29]. Therefore, in stage 1, we aim to explore whether acupuncture improves PDA by regulating the HPA via changes in the CRH, CORT, ACTH, and 5-HT levels.

To reduce bias, we will use a rigorous allocation method with blinded patients, evaluators, and statistical analysts. We will also use a new type of sham acupuncture tool to avoid the placebo effect. Therefore, this trial will potentially provide valid clinical evidence. However, this study has certain limitations. First, owing to the nature of acupuncture, the operator cannot be blinded in this study. However, we will ensure the blinding of the participants, evaluators, and statistical analysts. Second, although we strictly require no increase or decrease of the medication dosage of the participants during treatment, it is difficult to monitor the self-medication of patients. Third, although a strict sample size calculation was carried out for this study, the participants will be recruited from only one hospital, which may lead to a lack of representativeness of the data.

Whether a treatment measure is suitable for clinical application and promotion needs to be comprehensively considered from the aspects of clinical efficacy, cost and economic benefits, patient acceptance, and feasibility. Therefore, this study has two stages. Stage 1 of this study hypothesizes that patients with PDA receiving acupuncture will have a reduction in anxiety as a result of adjustments to the HPA on the basis of excluding the placebo effect. Stage 2 of this study hypothesizes that acupuncture to relieve PDA has the characteristics of high feasibility, fewer side effects, high patient compliance, and reasonable cost effectiveness. It will not add additional economic burden to patients and their families while providing a curative effect and improving the quality of life of patients. It is a treatment method with clinical promotion and application value. The second stage is a supplement to the first stage. We designed this two-stage trial to observe whether acupuncture has clinical application value in the treatment of PDA.

The findings of this study will help to validate the effects and mechanism of acupuncture as a complementary treatment method for PDA. The study has the potential to provide strong clinical evidence as well as new guidelines for the treatment of PDA.

## Figures and Tables

**Table 1:** The timeline of participant progress through stage 1.

	Baseline visit	Intervention (weeks 1–4)				Follow-up period
Time point	Week 0	Week 1	Week 2	Week 3	Week 4	Week 12
Signing consent	●					
General examination	●					
Inclusion/exclusion	●					
Grouping	●					
Medication status	●					●
Acupuncture/sham acupuncture	●	●	●	●	●	
HAMA	●				●	●
UPDRS	●				●	●
Hoehn–Yahr Rating Scale	●				●	●
SF-36 scale	●				●	●
Serological indicators	●				●	
Adverse events		●	●	●	●	
Safety assessment		●	●	●	●	

HAMA: Hamilton Rating Scale for Anxiety; UPDRS: Unified Parkinson's Disease Rating Scale.

**Table 2:** Timeline of the progress of the participant in stage 2.

	Baseline visit	Intervention (weeks 1–4)			
Time point	Week 0	Week 1	Week 2	Week 3	Week 4
Signing consent	●				
General examination	●				
Risk factor	●				
Medication status	●				
Safety eligibility	●				
Allocation	●				
Acupuncture + anti-Parkinson drugs + CMO		●	●	●	●
Anti-Parkinson drugs + CMO		●	●	●	●
Feasibility and universality					●
Cost effectiveness					●
HAMA	●				●
UPDRS	●				●
Hoehn–Yahr Rating Scale	●				●
SF-36 scale	●				●
Serological indicators	●				●
Adverse events		●	●	●	●
Safety assessment		●	●	●	●

CMO: clinical monitoring only; HAMA: Hamilton Rating Scale for Anxiety; UPDRS: Unified Parkinson's Disease Rating Scale.

## Data Availability

No data were used to support this study.
